# Analysis of Laparoscopic Sleeve Gastrectomy Learning Curve and Its Influence on Procedure Safety and Perioperative Complications

**DOI:** 10.1007/s11695-017-3075-x

**Published:** 2017-12-23

**Authors:** Piotr Major, Michał Wysocki, Jadwiga Dworak, Michał Pędziwiatr, Magdalena Pisarska, Mateusz Wierdak, Anna Zub-Pokrowiecka, Michał Natkaniec, Piotr Małczak, Michał Nowakowski, Andrzej Budzyński

**Affiliations:** 1 2nd Department of General Surgery, Jagiellonian University Medical College, Kopernika 21 St, 31-501 Krakow, Poland; 2 Centre for Research, Training and Innovation in Surgery (CERTAIN Surgery), Krakow, Poland; 3 Department of Medical Education, Jagiellonian University Medical College, Kopernika 21 St, 31-501 Krakow, Poland

**Keywords:** Laparoscopic sleeve gastrectomy, Learning curve, Surgical training, Clinical competence

## Abstract

**Purpose:**

Laparoscopic sleeve gastrectomy (LSG) has become an attractive bariatric procedure with promising treatment effects yet amount of data regarding institutional learning process is limited.

**Materials and Methods:**

Retrospective study included patients submitted to LSG at academic teaching hospital. Patients were divided into groups every 100 consecutive patients. LSG introduction was structured along with Enhanced Recovery after Surgery (ERAS) treatment protocol. Primary endpoint was determining the LSG learning curve’s stabilization point, using operative time, intraoperative difficulties, intraoperative adverse events (IAE), and number of stapler firings. Secondary endpoints: influence on perioperative complications and reoperations. Five hundred patients were included (330 females, median age of 40 (33–49) years).

**Results:**

Operative time in G1–G2 differed significantly from G3–G5. Stabilization point was the 200th procedure using operative time. Intraoperative difficulties of G1 differed significantly from G2–G5, with stabilization after the 100th procedure. IAE and number of stapler firings could not be used as predictor. Based on perioperative morbidity, the learning curve was stabilized at the 100th procedure. The morbidity rates in the groups were G1, 13%; G2, 4%; G3, 5%; G4, 5%; and G5, 2%. The reoperation rate in G1 was 3%; G2, 2%; G3, 2%; G4, 1%; and G5, 0%.

**Conclusion:**

The institutional learning process stabilization point for LSG in a newly established bariatric center is between the 100th and 200th operation. Initially, the morbidity rate is high, which should concern surgeons who are willing to perform bariatric surgery.

## Introduction

Laparoscopic sleeve gastrectomy (LSG) has become an attractive, one-stage, and bariatric procedure with promising short- and long-term treatment effects in morbidly obese patients [1, 2]. LSG is considered as one of the least technically challenging bariatric procedures. Along with high safety profile and good outcomes, it is often a sufficient reason for selecting LSG as the first-line treatment in newly established bariatric centers for gaining experience in weight loss surgery. The perioperative complication rate of LSG is estimated to be between 7 and 15%. The mortality risk of LSG ranges between 0.1 and 2% [3–5]. However, these rates are considered higher in low-volume bariatric centers and in newly established centers [6, 7]. In 1885, a German psychologist, Hermann Ebbinghaus, was the first to describe the learning curve process. A learning curve is currently defined as the amount of repetition required for a particular activity to establish an expert level of performance in this particular action [8]. With regard to surgical procedures, a learning curve is defined as the number of consecutive procedures required to become an expert in carrying out a specific operation. Most authors agree that the LSG learning curve is 50–100 procedures per surgeon [9, 10]. However, the amount of data regarding the LSG learning curve for the whole bariatric center—the institutional learning process and its effect on the bariatric treatment course—is scarce. As in most surgical procedures, the safety of the operation depends mainly on the experience of the team performing the procedure, not on the brilliant surgeon performing all of the operations. It can, therefore, be stated that the experience of the laparoscopic center and the entire team rather than individual surgeons translates into complication rate and outcomes. Therefore, with attendees and residents as main operators, this study aimed to analyze the stabilization point of the institutional learning process for bariatric team of six operators, including two attending surgeons and four surgery residents, in a newly established bariatric center. We also attempted to analyze the effect of this learning process on patients’ safety and the incidence of perioperative complications.

## Material and Methods

**Fig. 1:**
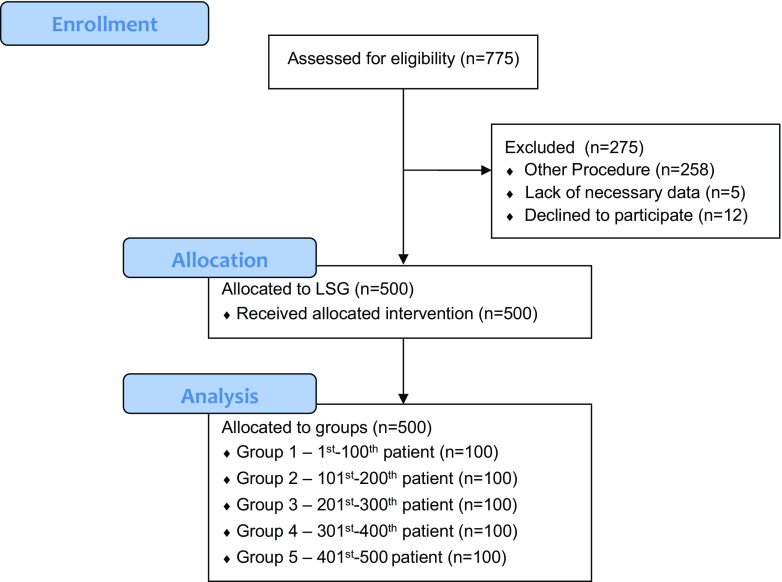
Study flow chart

We performed a retrospective analysis of prospectively collected data of patients who were submitted to surgical treatment of morbid obesity in the Second Department of General Surgery, Jagiellonian University Medical College (academic teaching hospital and tertiary referral center for general surgery). Criteria for surgical treatment were in accordance with the guidelines of the Metabolic and Bariatric Surgery Section of the Polish Surgical Society (i.e., body mass index [BMI] ≥ 35 kg/m^2^ with obesity comorbidities or BMI ≥ 40 kg/m^2^). In our study, we included 18–65-year-old patients, who agreed to voluntarily use their data in retrospective studies and were submitted to LSG as primary treatment for morbid obesity. Patients meeting the study inclusion criteria were divided into groups every 100 consecutive patients. The flow of patients through the study is shown in Fig. 1.

The bariatric team that performed the surgeries comprised of six operators, including two attending surgeons and four surgery residents in their third year of training (out of 6 years). In each case, one attending surgeon and one resident were scrubbed. Introduction of bariatric procedures was structured. Initially, the most experienced operator in laparoscopic surgery visited high-volume bariatric centers to participate in bariatric internships. Procedures then started in the department along with the introduction of a treatment protocol. Guest surgeons from high-volume bariatric centers proctored the learning process in the bariatric team in the first 15 cases during 2 weeks. Requirements for surgeons in training were acquirement of an appropriate theoretical background in bariatric surgery and prior experience in laparoscopic surgery (including intra-corporal sewing skills and structured training on simulators). Prior to training as an operator in the operating room, apart from gaining a theoretical background, surgeons needed to assist in a minimum of 15 LSGs. After meeting the requirements, the residents started operating as the first operator with the attending surgeon assisting with the patient. We defined the number of 100 operations as a basic unit for further analysis. To minimize bias, the patients’ care was standardized in accordance with the principles of the multimodal Enhanced Recovery after Surgery (ERAS) pathway, as described previously [11–13]. During preoperative counseling, patients aged over 50 years, with maximal preoperative BMI over 45 kg/m^2^, with obesity-related comorbidities (especially type 2 diabetes mellitus and gastroesophageal reflux disease, GERD), hiatal hernia, and previous gastric resections were factors indicating qualification for LRYGB, not for LSG in our facility. Routine endoscopy of the upper gastrointestinal tract was done with assessment of the incidence of hiatal hernia and esophageal, gastric or duodenal mucosa pathology. If there were symptoms of GERD and endoscopic findings corresponding with GERD, patient was not qualified to LSG.

With regard to the surgical technique, it was standardized as described previously [14].

The analyzed group was divided into subgroups according to the order of the procedure: G1, group number 1 (1st–100th LSG); G2, group number 2 (101st–200th LSG); G3, group number 3 (201st–300th LSG); G4, group number 4 (301st–400th LSG); and G5, group number 5 (401st–500th LSG).

The primary endpoint was determining the stabilization point of the LSG learning curve in each group using operative time, intraoperative difficulties, intraoperative adverse events, and the number of stapler firings. As secondary endpoints, the effects of the learning curve on perioperative complications and re-operation rate were assessed.

Directly after each procedure, every surgeon who performed the operation as the main operator was obligated to note down the intraoperative difficulties, which were defined as surgeon-reported obstacles during the operation. These obstacles were additional measures that were required to finish the procedure or those that significantly prolonged the procedure. These obstacles also included difficulty in achieving a sufficient working space, difficulty in proper setting of the stapler, intra-abdominal anatomy obstructing performance of the surgery, or the need for assistance from a supervisor. The definition of intraoperative difficulties is similar to that reported by other authors [15, 16]. Intraoperative adverse events were defined as any iatrogenic, adverse event during the operation, which was not derived from the standard LSG technique. Perioperative complications were defined as adverse events occurring within 30 days after the procedure. These events were classified according to the Clavien–Dindo classification [17]. Rhabdomyolysis was defined as an elevated serum creatine phosphokinase concentration of > 1000 U/L with a concomitant increase in myoglobin concentrations.

### Statistical Analysis

All data were analyzed with Statsoft Statistica version 12.0 PL (StatSoft Inc., Tulsa, OK, USA). The results are presented as mean ± standard deviation (SD), median and interquartile range, and odds ratios (ORs) with 95% confidence intervals (CIs) when appropriate. To assess statistical significance of qualitative data differences in subgroups, Pearson’s chi-square test and discriminant analysis were used. Quantitative data were analyzed with the Kruskal–Wallis ANOVA and post-hoc testing. Univariate logistic regression was used to calculate ORs with 95% confidence intervals. Results were considered statistically significant when the *p* value was less than 0.05.

### Patients

From April 2009 to October 2017, 775 patients were treated for morbid obesity at the Second Department of Surgery, Jagiellonian University Medical College, including 500 consecutive patients qualified to LSG. A total of 500 patients were included in the study [330 females, 170 males median age of 40 (33–49) years, median BMI of 44.84 (min. 34.01; max. 76.44) kg/m^2^]. Patients’ characteristics are shown in Table 1.

**Table 1:** Patients’ characteristics

Demography	Total	G1	G2	G3	G4	G5	*P* value
Females, *n* (%)	330 (66)	69	63	64	70	64	0.759
Males, *n* (%)	170 (34)	31	37	36	30	36	
Median age, years (IQR)	40 (33–49)	39 (32.5–47)	41 (33–48.5)	40 (33–49)	39 (32–47)	42 (34–50)	0.400
Median BMI at qualification, kg/m^2^ (IQR)	46.44 (42.91–51.37)	46.56 (44.10–50.85)	44.62 (41.98–49.22)	46.71 (42.73–51.37)	45.81 (42.76–52.73)	47.11 (43.94–54.65)	0.345
Median BMI on admission, kg/m^2^ (IQR)	44.84 (41.33–49.22)	45.48 (42.45–49.80)	43.10 (41.04–47.66)	45.49 (41.14–49.12)	44.01 (40.88–49.51)	45.0 (41.87–50.20)	0.143
Cardiovascular diseases, *n* (%)	61 (12.2)	15	16	12	12	6	0.226
Arterial hypertension, *n* (%)	320 (64)	59	71	62	55	73	*0.034*
Respiratory diseases, *n* (%)	95 (19)	22	14	22	15	22	0.364
Diabetes mellitus, *n* (%)	119 (24)	22	21	28	24	24	0.808
Dyslipidemia, *n* (%)	180 (36)	60	50	38	16	16	*< 0.001*
Fatty liver disease, *n* (%)	219 (44)	65	60	56	15	23	*< 0.001*

*G1* group number 1 (1st–100th LSG), *G2* group number 2 (101st–200th LSG), *G3* group number 3 (201st–300th LSG), *G4* group number 4 (301st–400th LSG), *G5* group number 5 (401st–500th LSG)

## Results

### Operative Time and First Operators

**Fig. 2:**
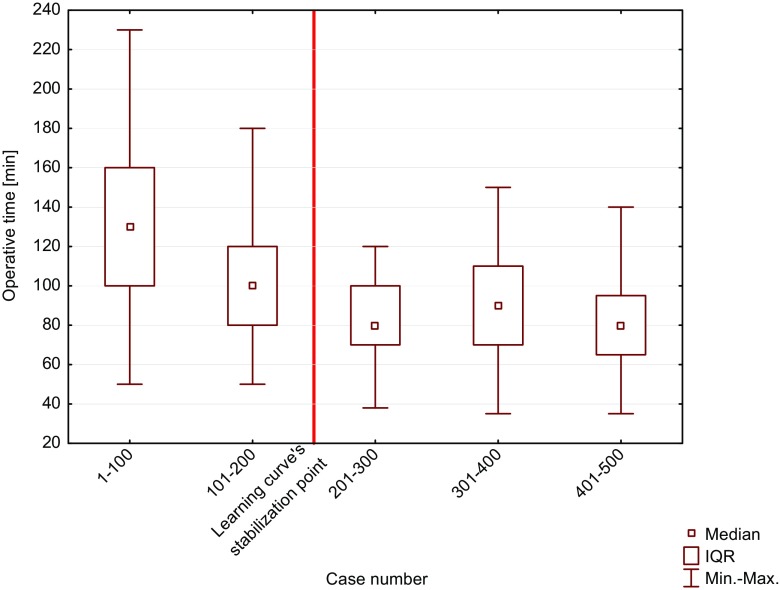
Graph illustrating operative time changes in institutional learning curve

The median operative time for LSG was 90 (70–120) minutes. The Kruskal–Wallis ANOVA showed that there was a significant difference in the median operative time between the groups (*p* < 0.001) (Table 2). Multiple comparisons of the median ranges for all groups showed significant differences between G1–G2 and G3–G5. Based on multiple comparisons of the median range tests, the LSG learning curve’s stabilization point was the 200th procedure for the whole newly established bariatric center (Fig. 2). During a study period, we observed a significant decrease in the number of operations performed by the attendees in favor of operations performed by the residents as first operators (Pearson’s test, *p* < 0.001).

**Table 2:** Operative time, operating surgeons, intraoperative difficulties, and intraoperative adverse events

	Total	G1	G2	G3	G4	G5
Median operative time (IQR)	90 (70–120)	130 (100–160)	100 (80–120)	80 (70–100)	90 (70–110)	80 (65–95)
First operator						
Attending	296 (59%)	91/100	87/100	53/100	35/100	30/100
Resident	204 (41%)	9/100	13/100	47/100	65/100	70/100
Intraoperative difficulties						
Total	23/500 (4.6%)	12/100 (12%)	2/100 (2%)	3/100 (3%)	2/100 (2%)	4/100 (4%)
Difficulty to achieve sufficient working space	6 (1.2%)	4	0	1	0	1
Difficulty of proper setting the stapler	7 (1.4%)	5	0	1	0	1
Intra-abdominal adhesions obstructing performance of the surgery	4 (0.8%)	2	0	0	1	1
Fatty liver disease obstructing the procedure	1 (0.2%)	0	1	0	0	0
Irreducible umbilical hernia	1 (0.2%)	0	1	0	0	0
Large umbilical hernia	1 (0.2%)	0	0	0	1	0
Required of the help of the mentor	1 (0.2%)	1	0	0	0	0
Difficulty to achieve sufficient hemostasis	2 (0.4%)	0	0	1	0	1
Intraoperative adverse events						
Total	9/500 (1.8%)	3/100 (3%)	0/100 (0%)	2/100 (3%)	2/100 (2%)	2/100 (2%)
Intraoperatively diagnosed leakage, supplied with the additional suturing	3 (0.6%)	3	0	0	0	0
Intraoperatively diagnosed leakage, supplied with setting of stent and drainage	1 (0.2%)	0	0	1	0	0
Excessive intraoperative blood loss	1 (0.2%)	0	0	1	0	0
Bleeding from liver/spleen	2 (0.4%)	0	0	0	1	1
Bleeding from stapler line	1 (0.2%)	0	0	0	1	0
Gastric content leak from resected part	1 (0.2%)	0	0	0	0	1

*G1* group number 1 (1st–100th LSG), *G2* group number 2 (101st–200th LSG), *G3* group number 3 (201st–300th LSG), *G4* group number 4 (301st–400th LSG), *G5* group number 5 (401st–500th LSG)

### Conversions

All procedures were completed laparoscopically.

### Intraoperative Difficulties

Intraoperative difficulties were reported during 23 (4.6%) procedures (Table 2). Using the group number as a factor in univariate logistic regression, the OR for the occurrence of intraoperative difficulties diminished with every 100 performed operations (OR, 0.68; 95% CI, 0.49–0.94; *p* = 0.019) and affected, respectively, in G1, 12 patients (12%); G2, two (2%); G3, three (3%); G4, two (2%); and G5, four (4%). According to Pearson’s chi-square test, there was a significant difference in the occurrence of intraoperative difficulties among the groups (*p =* 0.003). Based on intraoperative difficulties, stabilization point was at the 100th procedure in discriminant analysis.

### Intraoperative Adverse Events

Intraoperative adverse events were observed during nine (1.8%) operations (Table 2). In G1, they occurred in three patients, G2—none, G3—three patients, G4—two patients, and G5—two patients. Due to lack of variability, this parameter could not be used for estimating stabilization point (*p* = 0.607).

### Number of Stapler Firings

The median number of stapler firings in the groups was the following: G1, five (4–5); G2, four (4–5); G3, four (4, 5); G4, four (4, 5); and G5, five (4, 5). The Kruskal–Wallis ANOVA showed a significant difference in the number of stapler firings among the groups (*p* < 0.001). Multiple comparisons of the median and range of stapler firings for all groups showed that G1 and G5 differed from G2 and G4.

### Perioperative Morbidity

Perioperative complications were diagnosed in 29 (7.6%) patients (Table 3). During the study period, we diagnosed 13 patients with perioperative complications of Clavien–Dindo class I, 4 patients with class II, 10 patients with class III, and 2 patients with class IV. Detailed characteristic of perioperative morbidity regarding Clavien–Dindo scale is presented in Table 3. When we used group number as a factor in the univariate logistic regression model, the OR of perioperative morbidity significantly decreased with every 100 performed procedures (i.e., with every consecutive group) (OR, 0.66; 95% CI, 0.50–0.89; *p* = 0.006). The morbidity rates in the groups were G1, 13%; G2, 4%; G3, 5%; G4, 5%; and G5, 2%, respectively. Pearson’s chi-square test showed that there was a significant difference in the occurrence of morbidity among the groups (*p* = 0.011). Based on perioperative morbidity, the learning curve stabilized at the 100th procedure in discriminant analysis.

**Table 3:** Perioperative (≤ 30 days) morbidity regarding Clavien–Dindo scale and reoperations

C-D	Complications	No. (%)	G1	G2	G3	G4	G5
	Total	29/500 (7.6%)	13/100 (13%)	4/100 (4%)	5/100 (5%)	5/100 (5%)	2/100 (2%)
4b	Cardiorespiratory failure (ICU stay)	1 (0.2%)	0	1	0	0	0
4a	Pulmonary embolism (thrombolysis)	1 (0.2%)	0	0	0	0	1
3b	GI leak (relaparoscopy)	4 (0.8%)	3	1	0	0	0
	Operation site bleeding (relaparoscopy)	4 (0.8%)	0	1	2	1	0
	Wound infection (open drainage)	1 (0.2%)	0	0	0	1	0
3a	GI stricture (tube placement)	1 (0.2%)	0	0	1	0	0
2	Varicella infection	1 (0.2%)	0	0	1	0	0
	TIA	1 (0.2%)	0	0	0	1	0
	Superior mesenteric vein thrombosis (thrombolysis)	1 (0.2%)	0	0	0	1	0
	Renal colic	1 (0.2%)	0	0	0	0	1
1	Delayed gastric emptying due to temporary stricture *	5 (1%)	2	1	1	1	0
	Dehydration*	1 (0.2%)	1	0	0	0	0
	Prolonged drainage	1 (0.2%)	1	0	0	0	0
	Rhabdomyolysis	6 (1.2%)	6	0	0	0	0
	Reoperations	8 (1.8%)	3	2	2	1	0

*1 patient was diagnosed with both complications

*C-D* Clavien-Dindo classification grade, *G1* group number 1 (1st–100th LSG), *G2* group number 2 (101st–200th LSG), *G3* group number 3 (201st–300th LSG), *G4* group number 4 (301st–400th LSG), *G5* group number 5 (401st–500th LSG), *TIA* transient ischemic attack

### Reoperations

Reoperations were necessary in eight (1.8%) patients. Pearson’s chi-square test did not reveal significant differences of reoperations in groups (*p* = 0.508). It was also nonsignificant in univariate logistic regression model (*p* = 0.344). Basing on reoperations, this parameter could not be used for setting stabilization point.

### Mortality

None of the patients died during the 30-day perioperative period.

## Discussion

Bariatric surgery in Poland is still a developing discipline. We describe a 9-year experience of LSG in the Second Department of General Surgery, JUMC [18]. We aimed to assess our institutional learning process for LSG and the effect of this process on safety and perioperative complications.

Operative time is frequently regarded as a descriptive parameter for evaluating the learning curve. In our study, the operative time was an average of 90 min. Zacharoulis et al. assumed that one of the main determinants of an LSG learning curve’s stabilization point is the operative time and estimated that 68 consecutive procedures are essential to finish training [19]. Daskalakis et al. observed a significant difference in surgical duration between the first and second halves of their 230 consecutive LSGs [20]. A learning curve’s stabilization point is usually derived from the literature and tested for individual operators to achieve expertise. In our study, we to estimate the stabilization point for the whole department by testing the median and range within consecutively operated groups of 100 patients. In our study, the institutional learning process’ stabilization point was the 200th procedure.

We reported intraoperative difficulties during 23 (4.6%) procedures. In our study, the OR of the occurrence of intraoperative difficulties decreased with every 100 performed LSGs. Intraoperative difficulties were mainly related to a narrow operative space (six cases) and to proper stapler setting difficulty (seven cases). Unfortunately, we could not find any previously published studies regarding intraoperative difficulties, which are important parameters for assessment of the learning curve [21, 22]. Intraoperative difficulties were self-reported by surgeons and defined as obstacles requiring additional measures during the surgery. We estimated that the intraoperative difficulty rate stabilized after 100 procedures.

In our study, intraoperative adverse events, defined as harmful events related to surgery, occurred during nine (1.8%) procedures, and due to lack of variability, they could not be used for estimation of the institutional learning process’ stabilization point. There were four cases (0.8%) of intraoperatively diagnosed gastrointestinal leak. Similarly, Rubin et al. reported that intraoperative adverse events occurred during 3.33% of 120 LSGs. They found one case of intraoperatively diagnosed gastric leak, two cases of short gastric vessel bleeding, and one case of probe immobilization in the staple line [23]. Braghetto et al. reported a rate of 14% for intraoperative adverse events after introduction of LSG in a series of 50 LSGs (four cases of intraoperatively diagnosed gastric leak, one case of short gastric vessel bleeding, one case of liver capsule perforation with a retractor, and one case of probe immobilization in the staple line) [24].

After 100 procedures, the amount of stapler firings decreased by one firing but then in the last 100 patients, it increased by one. Kaska et al. found that, to avoid adverse events and complications, adequately choosing the cartridge and over-sewing the staple line are essential but the number of stapler firings was not analyzed [16]. We believe that this increase comes just from taking adequate measures to operative conditions. Our beliefs are supported by lack of increase in intraoperative adverse events and complications.

The conversion rate for LSG ranges from 1.05 to 1.85% [25]. Despite these rates, in our study, all procedures were completed laparoscopically.

The LSG learning curve’s stabilization point can also be estimated using perioperative morbidity. In our study, perioperative morbidity was 7.6%. An almost identical rate of the LSG learning curve was described by Zacharoulis et al. (7.8%) [19]. Our result is satisfactory compared with the reported a major morbidity rate of 12.1% (0–29%) in a systematic review by Shi et al. [26]. Regardless of implementation of the ERAS protocol along with introduction of LSG, we observed a relatively high perioperative morbidity of 13% in the first 100 patients. Morbidity then decreased to 4% in the rest of the consecutive patients. Discriminant analysis enabled us to set the LSG learning curve’s stabilization point based on morbidity at the 100th LSG. Zachariah et al. showed that the perioperative complication rate in the first 50 patients was significantly higher than that in the last 178 patients (8 vs. 1.6%, *p* = 0.02) [27]. Casella et al. showed that perioperative morbidity was significantly decreased after the 88th LSG [28].

In our study, eight (1.8%) patients required reoperations. This reoperation rate was significantly smaller than the rate reported by Daskalakis et al. (7.4%) [20]. In our study, due to low variability of reoperation rate, we could not draw conclusions from this parameter.

None of the patients died during the 30-day perioperative period. This finding is similar to that by Casella et al. [28] and Trastulli et al. [29]. However, Zacharoulis et al. showed that the mortality rate was 0.98% [19]. Taking into consideration, the mortality of LSG as reported in a systematic review by Shi et al. (0–3.3%), the lack of morality in our study on the course of the learning curve is satisfactory [26].

Usually, a learning curve is evaluated using selected parameters for particular surgeon. We assessed the institutional learning process using intraoperative difficulties, perioperative morbidity, and operative time, while obtaining accurate results. Operative time of the first 200 procedures was significantly longer than that of the next 300 surgeries. Based on this, the institutional learning process stabilization point was the 200th procedure for the whole newly established bariatric center. Intraoperative difficulties in the first 100 patients (12%) were significantly more prevalent than that in the next 400 procedures (2.75%). Based on intraoperative difficulties, the institutional learning process’ stabilization point was at the 100th procedure. Finally, the postoperative morbidity rate in the first 100 patients of 13% was much higher than that in the next 400 cases (4%). Based on morbidity, the institutional learning process was stabilized at the 100th procedure. Summarizing all endpoints that enabled to assess stabilization point, we proposed that the institutional learning process stabilized between the 100th and 200th case.

This study has several limitations. Our institution is a referral center for general surgery and a teaching hospital. Therefore, our results probably cannot be extrapolated to all hospitals. Additionally, this was a retrospective study. A prospective study should be designed to confirm these findings. Finally, the bariatric team consisted of surgeons of varying levels of experience, which may have affected the results. A larger amount of operated patients would enable the use of all of the parameters we attempted to test for describing the learning curve. Further research should be performed using intraoperative difficulties as a self-reported assessment for evaluation of the learning curve process.

## Conclusion

The institutional learning process’ stabilization point for LSG in a newly established bariatric center is between the 100th and 200th operation. LSG, which is carried out with a learning curve, does not affect the safety of the procedure in terms of intraoperative adverse events. Initially, with introduction of LSG, the morbidity rate is high, which should concern surgeons who are willing to start performing bariatric surgery.
